# An audit of central venous catheter insertion and management practices in two French university hospitals

**DOI:** 10.1007/s10096-024-04906-8

**Published:** 2024-07-25

**Authors:** Nagham Khanafer, Sophie Gardes, Nathalie De-Santis, Céline Liard, Florian Deschamps, Pauline Verbist, Stephane Nancey, Eddy Cotte, Olivier Martin, Laurent Argaud, Anne Claire Lukaszewicz, Philippe Vanhems

**Affiliations:** 1 Service d’Hygiène Epidémiologie et Prévention, Hôpital Edouard Herriot, Hospices Civils de Lyon (HCL), Lyon, France; 2 PHE3ID team, Centre International de Recherche en Infectiologie, Inserm U1111, CNRS UMR5308, ENS de Lyon, Lyon 1 University, Lyon, France; 3 Service d’Hygiène, Epidémiologie et Prévention, Hôpital Lyon Sud, HCL, Pierre Bénite, France; 4 Becton Dickinson and Company, Le Pont-De-Claix, France; 5 Department of Hepato-Gastroenterology, Hôpital Lyon, Sud, HCL, Pierre Bénite, France; 6 Department of surgery, Hôpital Lyon Sud, HCL, Pierre Bénite, France; 7 Intensive Care Department, Hôpital Edouard Herriot, HCL, Lyon, France

**Keywords:** Audit, Central venous catheter, Infection control and prevention, Practices

## Abstract

**Objective:**

To assess the compliance with French guidelines for the prevention of central venous catheter (CVC)-related infections in two university hospitals.

**Methods:**

An observational audit was conducted in 7 wards using a digital tool.

**Results:**

The prerequisite of hand hygiene (HH) were respected by 90% of health-care worker; 86% performed HH prior to equipment preparation and 59% repeated it prior to infusion. Wearing gloves when necessary and rinsing were respected in 46.7% and 75.6% of the observations.

**Conclusion:**

Findings showed an acceptable level of adherence to recommended practices for CVC management. However, barriers of unrespect evidence-based recommendations need to be investigated in depth.

## Introduction

Central venous catheters (CVCs) are commonly used in hospitalized patients, particularly in the intensive care unit (ICU), and in units where long-term administration of cancer chemotherapies and parenteral nutrition are necessary [1]. Despite their huge benefits, they also have complications (thrombotic, mechanical,…) of various severities, including infection.

In France, the prevalence survey of 2022 showed that 3.2% of hospitalized patients had a CVC compared to 2.3% in 2017, which is probably related to an increase in vulnerability factors of patients [2]. In the Eurobact 2 study, CVC-related bacteremia accounted for 26.4% of bacteremia cases in the ICU and was associated with a mortality rate of 24.7% [3].

Outside specific vascular units, operating room, or interventional radiology department, CVCs can be inserted in the ICU room under aseptic conditions that are more difficult to control, even though it is a frequent and “protocoled” procedure. The daily manipulation of catheter multiple times per day for the administration of fluids, drugs, and blood products, and the frequency of connection and disconnection procedures pose significant challenges in controlling infectious risks associated with CVC. The French Society of Hospital Hygiene (SF2H) has published its recommendations for the prevention of this risk, which have been incorporated shortly thereafter into institutional protocols for the management of these devices [4–6].

This implementation project aimed to assess compliance with French guidelines for the prevention of CVC-related infections in two university hospitals.

## Methods

A multicenter collaborative audit was conducted between the infection control teams (ICT) of Edouard Herriot Hospital and Lyon Sud Hospital, two university hospitals of Hospices Civils de Lyon, Lyon, France and Becton Dickinson (BD), a company producing catheters. Two trained nurses from BD (previously working in hospital) completed separately the observations in 7 wards: ICU (*n* = 4) and surgery (*n* = 3). The targeted units had not been audited in the last five years. The audit grid (100 criteria) was developed by the ICT and BD based on the recommendations of the French Society of Hospital Hygiene [4–6]. Signature Solutions™, a digital tool developed by BD, was used to capture the observations in real time and to allow their analysis. Descriptive analyses were performed to describe the percentage of compliance with national recommendations. The data were reported as the number of observations and not as the number of professionals. A feedback session was held for each unit, attended by the health-care worker (HCW) manager, doctors, and/or technical nurses, with the unit’s hygiene correspondents present whenever feasible.

The implementation of the audit was validated by the establishment’s legal experts and the Data Protection Officer.

## Results

**Fig. 1:**
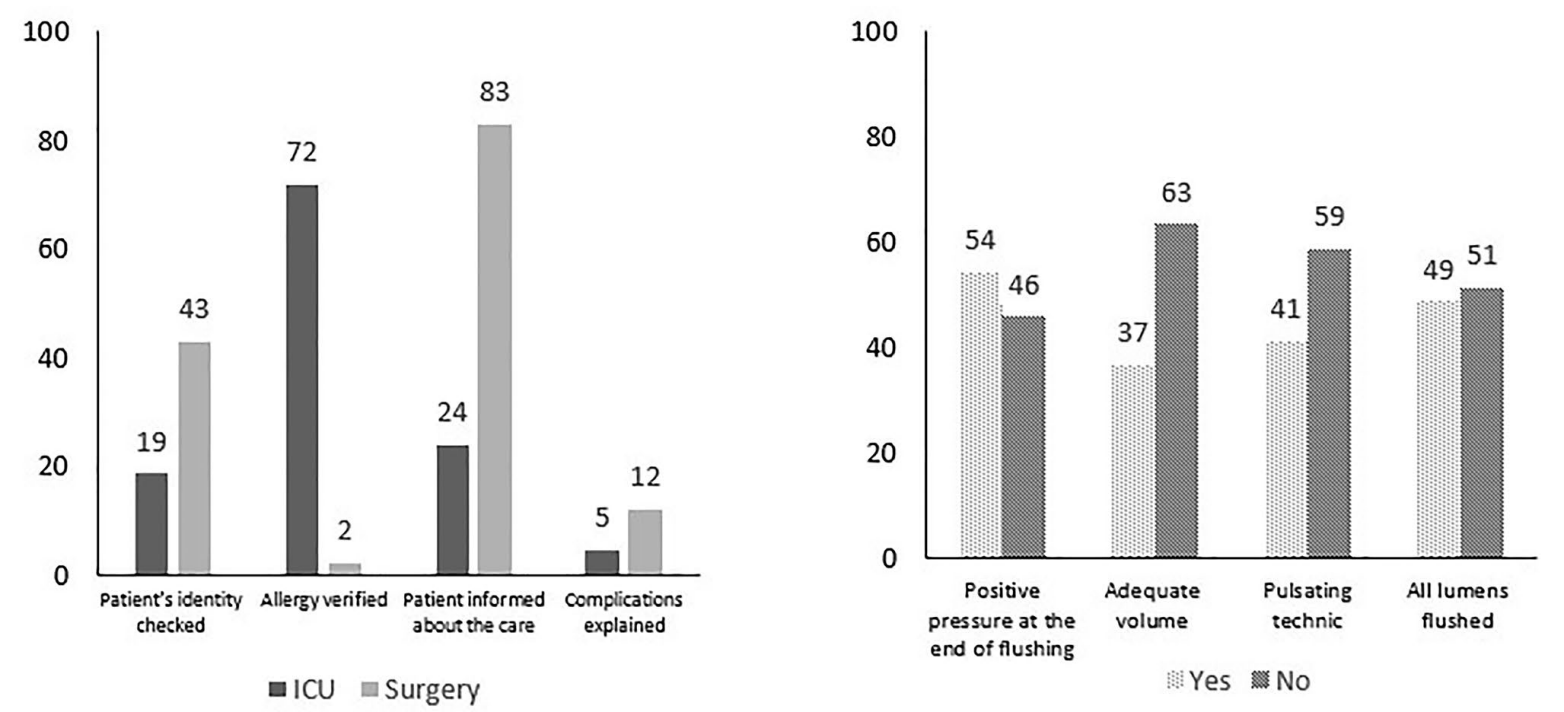
Percentage of compliance. (**A**) Percentage of compliance of care safety by participating department. (**B**) Percentage of compliance of catheter flushing.

A total of 90 observations of CVCs maintenance were carried out between July and November, 2021 in 4 ICUs (*n* = 48) and 3 surgical units (*n* = 42). To complete these observations, 12 working days were necessary. In 40% of observations, allergies were assessed, while in about 12% of observations, complications were explained (Fig. 1A). Figure 1 A shows also that patient’s identity was verified in 31% of observations and the procedure was explained to the patient in 54% of observations indicated that the procedure was explained to the patient.

The compliance with the criteria used to evaluate the rinsing techniques, including the use of an appropriate volume (= 10 mL), a pulsating technic, flushing of all lumens, and the application of positive pressure at the end of the procedure, is illustrated in Fig. 1B. is Compliance with hand hygiene prerequisites (short-sleeved uniform, no jewelry, and short nails without varnish or artificial nails), hand hygiene indications, checking product expiration dates, and handling lines are described in Table 1.

**Table 1:** Description of compliance regarding hand hygiene and CVC handling

	Compliant observations, *n* (%)
Hand hygiene prerequisites^(1)^, *n* = 90	81 (90)
Hand hygiene before the preparation of materials, *n* = 88	76 (86)
Repeated hand hygiene before infusion/handling lines, *n* = 84	50 (59)
Checking product expiration dates, *n* = 90	13 (14)
Handling lines with gauze and alcoholic antiseptic^(2)^, *n* = 90	75 (83)
Wearing gloves when recommended^(3)^, *n* = 90	42 (46)
Rinsing performed, *n* = 90	68 (75)

^(1)^ Short-sleeved uniform, no jewelry, and short nails without varnish or artificial nails

^(2)^ Using gauze impregnated by an alcoholic antiseptic when the catheter lumen is open (change of extension with or without valve) and for administration of medication and blood sampling

^(3)^ Sterile gloves are recommended for the insertion of CVC or when the lumen of CVC is open (see (1)). Alternatively, non-sterile gloves are recommended for blood sampling

## Discussion

CVCs are widely used in the ICU, as well as in other surgical and medical departments. Compliance with recommendations can help reduce the risks associated with these devices, particularly the risk of infection, which is largely preventable [7]. Our audit’s findings revealed a certain degree of variation in adherence to current protocols. More than 70% of the observations showed compliance with good practices concerning the use of gauze and alcoholic antiseptic for line handling, as well as the performance of rinsing at the end of the procedure. However, some practices were not in line with evidence-based recommendations, particularly hand hygiene, rinsing procedures, asepsis, etc. Similar results have been reported in the literature [8, 9].

The use of gauze and alcoholic antiseptic for line handling preceded by hand hygiene is crucial for preventing the risk of infection. Numerous studies have demonstrated the value of antiseptic-impregnated caps for better control of this risk [10–12]. The cost is undoubtedly high; however, it provides enhanced prevention, particularly in hospitals/wards experiencing problems with the application of standard precautions.

Mechanical complications of CVCs can be prevented by the implementation of the pulsed flushing technique, which also helps to prevent drug incompatibilities and catheter occlusion during use [13]. Our results show that pulsed rinsing method is utilized in only 41% of observations. Therefore, this represents an essential aspect that must be addressed in our action plan.

Differences in practice were observed between ICU and non-ICU wards, particularly in terms of the “procedure explained” criteria, which were linked to the patient’s cognitive (e.g. Sedation, coma,.) capacity during the CVC care, as well as in terms of allergy testing and identity verification.

The ICT provided feedback on the results to raise awareness among participating teams concerning the importance of effective CVC management, identifying areas in need for improvement, and establishing training programs to be adapted to each specialty in terms of duration and methodology (such as instructor-based education, virtual instructor-led training, scenario-based learning, simulation-based learning, e-learning.). This feedback was an opportunity to remind the importance of ongoing training in this area, given the development of medical devices and recommendations.

The surveillance of infections on CVC outside ICU and/or hospital is complex. The short duration of hospitalization, the practice of patients maintaining central lines outside of hospitals is increasing; the difficulty to follow-up post discharge can underestimate the burden of these infections [14]. HCWs are less well trained and not sufficiently aware about the risks associated with these devices, which is why observations in these sectors are particularly important [15].

A prospective study combining audit, training, and patient monitoring was planned for 2024 in the 4 ICUs that took part in this audit. The following areas will be proposed to improve practices: knowledge of the different types of CVC, principles of use, awareness of related risks, the rinsing process, antisepsis of the skin, etc.

## Conclusion

Our results indicated a satisfactory level of compliance with National guidelines for the management of CVC. Nevertheless, it is essential to investigate the barriers that impede the respect of evidence-based recommendations. This study is part of a dynamic approach to enhance the quality and safety of care. Evaluations of professional practices, aim to reduce discrepancies between what is done and what should be done according to the guidelines and recommendations. This collaboration with an industrial company and the use of a digital tool significantly minimized the time required to assess CVC management and compare them to current protocols.

## Data Availability

No datasets were generated or analysed during the current study.
